# Epigenetic stress memory: A new approach to study cold and heat stress responses in plants

**DOI:** 10.3389/fpls.2022.1075279

**Published:** 2022-12-08

**Authors:** Muthusamy Ramakrishnan, Zhijun Zhang, Sileesh Mullasseri, Ruslan Kalendar, Zishan Ahmad, Anket Sharma, Guohua Liu, Mingbing Zhou, Qiang Wei

**Affiliations:** ^1^ Co-Innovation Center for Sustainable Forestry in Southern China, Bamboo Research Institute, Key Laboratory of National Forestry and Grassland Administration on Subtropical Forest Biodiversity Conservation, College of Biology and the Environment, Nanjing Forestry University, Nanjing, Jiangsu, China; ^2^ Bamboo Industry Institute, Zhejiang A&F University, Hangzhou, Zhejiang, China; ^3^ School of Forestry and Biotechnology, Zhejiang A&F University, Hangzhou, Zhejiang, China; ^4^ Department of Zoology, St. Albert’s College (Autonomous), Kochi, Kerala, India; ^5^ Helsinki Institute of Life Science HiLIFE, Biocenter 3, University of Helsinki, Helsinki, Finland; ^6^ National Laboratory Astana, Nazarbayev University, Astana, Kazakhstan; ^7^ State Key Laboratory of Subtropical Silviculture, Bamboo Industry Institute, Zhejiang A&F University, Hangzhou, Zhejiang, China; ^8^ Zhejiang Provincial Collaborative Innovation Center for Bamboo Resources and High-Efficiency Utilization, Zhejiang A&F University, Hangzhou, Zhejiang, China

**Keywords:** epigenetics, DNA methylation, chromatin remodelling, histone modifications, stress memory, somatic memory, transgenerational memory, intergenerational memory

## Abstract

Understanding plant stress memory under extreme temperatures such as cold and heat could contribute to plant development. Plants employ different types of stress memories, such as somatic, intergenerational and transgenerational, regulated by epigenetic changes such as DNA and histone modifications and microRNAs (miRNA), playing a key role in gene regulation from early development to maturity. In most cases, cold and heat stresses result in short-term epigenetic modifications that can return to baseline modification levels after stress cessation. Nevertheless, some of the modifications may be stable and passed on as stress memory, potentially allowing them to be inherited across generations, whereas some of the modifications are reactivated during sexual reproduction or embryogenesis. Several stress-related genes are involved in stress memory inheritance by turning on and off transcription profiles and epigenetic changes. Vernalization is the best example of somatic stress memory. Changes in the chromatin structure of the *Flowering Locus C* (*FLC*) gene, a MADS-box transcription factor (TF), maintain cold stress memory during mitosis. *FLC* expression suppresses flowering at high levels during winter; and during vernalization, B3 TFs, cold memory *cis*-acting element and polycomb repressive complex 1 and 2 (PRC1 and 2) silence *FLC* activation. In contrast, the repression of *SQUAMOSA promoter-binding protein-like* (*SPL*) TF and the activation of *Heat Shock* TF (*HSFA2*) are required for heat stress memory. However, it is still unclear how stress memory is inherited by offspring, and the integrated view of the regulatory mechanisms of stress memory and mitotic and meiotic heritable changes in plants is still scarce. Thus, in this review, we focus on the epigenetic regulation of stress memory and discuss the application of new technologies in developing epigenetic modifications to improve stress memory.

## Introduction

Extreme temperatures, such as cold and heat in the wake of climate change, significantly impact plant productivity by affecting gene expression. Therefore, plants memorize a stressful experience for a certain period of time and use their stress memory, such as transcriptional, somatic, intergenerational and transgenerational stress memory to increase their survival, when they are exposed to such conditions a second time (Xie et al., 2021; Brenya et al., 2022; Gallusci et al., 2022). Since plants do not have a brain, plant stress memory is completely different from human and animal memory. It is regulated by epigenetic changes and plays an important role in gene regulation by sensing and responding to cold and heat stress. This leads to the ability to develop appropriate physiological and molecular changes. In addition, epigenetically regulated stress memories are inherited through the next generations (Friedrich et al., 2019; Nguyen et al., 2022; Saeed et al., 2022; Sharma et al., 2022).

Salicylic acid and the downstream signalling protein are involved in such a memory process (Wu et al., 2012). During this process, the transcription of salicylic acid-responsive genes is activated. Elevated concentrations of salicylic acid result in changes in chromatin modification for these target genes. For example, salicylic acid-induced H3 acetylation, H4 acetylation and H3K4 methylation were increased at the promoter regions of stress-related genes (Butterbrodt et al., 2006), but chromatin modifications are dynamic when they are exposed to stress (Bhadouriya et al., 2021). Thus, changes in histone modifications either positively or negatively influence the expression of stress memory genes (Liu et al., 2022a). Constitutive photomorphogenesis 5A (CSN5A) regulates heat stress memory by increasing H3K4me3 in memory genes, *APX2* and *HSP22*, after heat stress (Singh et al., 2021). H3K4me3 also regulates transcriptional memory of stress-related genes such as *Fa-heat shock protein 17.8 Class II (FaHSP17.8-CII)* (Bi et al., 2021). Chromatin protein BRUSHY1 (BRU1) is required to maintain the transcriptional induction of heat stress memory genes (Brzezinka et al., 2019). In contrast, the changes of H3K27me3 of *Flowering Locus C* (*FLC*) gene maintain cold stress memory (Xie et al., 2021). This suggests that chromatin structure and accessibility play an important role in gene expression and are often associated with epigenetic regulations (epigenetic code) such as histone variants, histone post-translational modifications (PTMs), DNA methylation and some non-coding RNAs (Duan et al., 2018; Chang et al., 2020; Joseph and Young, 2022). In addition, histone modification and replacement of histones by histone variants are responsible for the inheritance of stress memory (Borg et al., 2021).

Nevertheless, plants have their limitations in adapting to stress conditions (Mahawar and Shekhawat, 2018; Khator et al., 2020), and it is still unclear to what extent these modifications contribute to the stability of systemic acquired stress resistance in terms of improved stress memory. Therefore, identifying epigenetic codes of plant stress memory responsible for stress responses is of great importance to developing stress-tolerant crops. However, the integrated view of epigenetic changes and their associations with stress memory and subsequent gene expression in plants is still scarce, except for a few reviews (Jacques et al., 2021; Xie et al., 2021; Gallusci et al., 2022; Liu et al., 2022a; Sharma et al., 2022). These reviews provided a general overview of stress memory during abiotic stress and raised many questions, such as how epigenetic stress memory allows plants to integrate information from previous stress conditions, how epimutations modulate stress memory, how memory types are maintained, etc. However, none focused exclusively on memory during cold and heat stress. In this review, we, therefore, discuss the histone modification and DNA methylation regulating chromatin structure and cold and heat stress memories. In addition, we focus on the application of emerging technologies in epigenetic modification and stress memory for a better future.

## Histone and DNA modifications

Modified by various PTMs such as acetylation, methylation, phosphorylation, etc., histone proteins regulate gene expression, DNA damage repair, DNA replication, and recombination (Han et al., 2022; Millán-Zambrano et al., 2022). Transcriptional activation or repression depends on which histone residues are modified and the type of PTMs (Sirko et al., 2022). For example, the methylation of lysine 4 and lysine 36 on histone H3 is associated with gene activation, while the methylation of lysine 9 and lysine 27 is associated with gene silencing. However, the histone code hypothesis states that different histone modifications in one or more tails sequentially or in combination become a histone code that can be read by other proteins to make different changes in gene expression (Millán-Zambrano et al., 2022). The histone code is responsible for storing plant epigenetic memory (Borg et al., 2020). Histone codes with specific combinations of histone variants and PTMs of the N-tail of histone proteins provide a large number of possibilities for nucleosome composition (Brewis et al., 2021), thereby playing an important role in regulating the expression of the genetic code. Similar to histone modification, DNA methylation, a conserved heritable epigenetic mark, influences nuclear gene expression and genome stability, thus regulating many biological processes. Moreover, changes in DNA methylation led to abnormalities in plant development (Lang et al., 2017). In this review, we discuss the epigenetic signatures that regulate plant stress memory under cold and heat stress conditions.

## Regulation of cold stress in plants

Chilling (0 to 15°C) and freezing (<0°C) are two distinct types of cold stresses, which decrease plant growth by inducing photosynthesis-associated damages, chlorosis, unregulated apoptosis, loss of cell membrane and protein fluidity etc. (Banerjee et al., 2017). Several plants increase freezing tolerance (cold acclimation) when they are exposed to low non-freezing temperatures (Adhikari et al., 2022). However, several important crops like rice, maize, soybean, cotton and tomato are sensitive to cold stress, and they are incapable of acclimatising to cold stress, when ice forms in their tissue. In contrast, many plant species require exposure to prolonged cold periods so that they can flower during the next spring under favourable conditions. This is called vernalization (Antoniou-Kourounioti et al., 2021). Plant cold acclimatisation gradually decreases after vernalization, one of the best examples of plant epigenetic regulation to adapt to environmental cues (Luo and He, 2020; Kang et al., 2022). Genetic and molecular analyses have shown that epigenetic marks play a major role in vernalization, whose response is based on an epigenetic memory of plants (Sharma et al., 2022). Histone modifications and DNA methylation are the major epigenetic marks of plants that can be identified during cold stress (Banerjee et al., 2017), but it is still unknown whether the activation of epigenetic marks and stress memories differ in response to chilling and freezing.

## Histone modifications during cold stress

Studies have revealed that histone modifications, such as methylation and acetylation, change under cold stress and target different molecular mechanisms, including cold-responsive genes that can be genetically manipulated (Bhadouriya et al., 2021; Kidokoro et al., 2022). Histone demethylases, *Jumonji C domain-containing* genes (*JMJC5*) regulate demethylation at the lysine or arginine residues of the histone and respond to circadian clock. *JMJC5* in *Medicago truncatula* was observed with the cold-dependent alternative splicing, which was reversed when plants returned to normal conditions, suggesting that the cold-dependent alternative splicing is associated with the epigenetic regulation of cold stress (Shen et al., 2016). Genomic regions containing active *cis*-acting elements, identified as DNase I hypersensitive sites (DHSs) in potatoes using chromatin analysis, play significant roles in regulating the cold stress response. Cold-stress induced DHSs are enriched in genic regions and regulate the expression of cold-responsive genes associated with bivalent histone modifications, H3K4me3 and H3K27me3 (Zeng et al., 2019).

In the regions of stress memory genes, both H3K4me3 and H3K27me3 were enriched, suggesting that cold-responsive genes increase chromatin accessibility under cold stress (Liu et al., 2014). In *Arabidopsis*, H3K27me3 decreases in two cold-responsive genes *COR15A* and *ATGOLS3* and gradually promotes the expression of these genes under increasing cold stress. When cold-exposed plants returned to normal conditions, the plants retained decreased H3K27me3 in *COR15A* and *ATGOLS3* and or histone replacement. Nevertheless, the expression of these two genes was not increased, suggesting that H3K27me3 does not inhibit gene activation but that H3K27me3 was inherited through cell division to serve as a memory marker for cold stress (Kwon et al., 2009). Polycomb repressive complex 2 (PRC2) increases H3K27me3 levels to suppress *FLC* activation during vernalization and maintain cold stress memory in chromatin regions in *Arabidopsis*, intimating that different stages of polycomb silencing are required to maintain epigenetic cold memory in *Arabidopsis* (Yang et al., 2017).

Histone acetylation normally promotes gene expression. Under cold stress, histone H3 acetylation promotes the activation of *COR* genes, and histone acetyltransferase HAC1 is necessary for a stress memory and enhances cold memory (Friedrich et al., 2019). Cold exposure also promotes histone deacetylation. *Zea mays* was observed with the upregulation of histone deacetylases (HDACs) and led to deacetylation at the lysine residues on the histone subunits H3 and H4 during cold acclimatisation. Thus, HDACs’ repression reduces the expression of cold-responsive genes *ZmDREB1* and *ZmCOR413* in *Zea mays* (Hu et al., 2011). In HDACs, three major families have been identified such as reduced potassium dependency protein 3 superfamily (RPD3/HDA1), silent information regulator protein 2 family (SIR2) and histone deacetylase 2 families (HD2) (Pandey et al., 2002).

**Figure 1:**
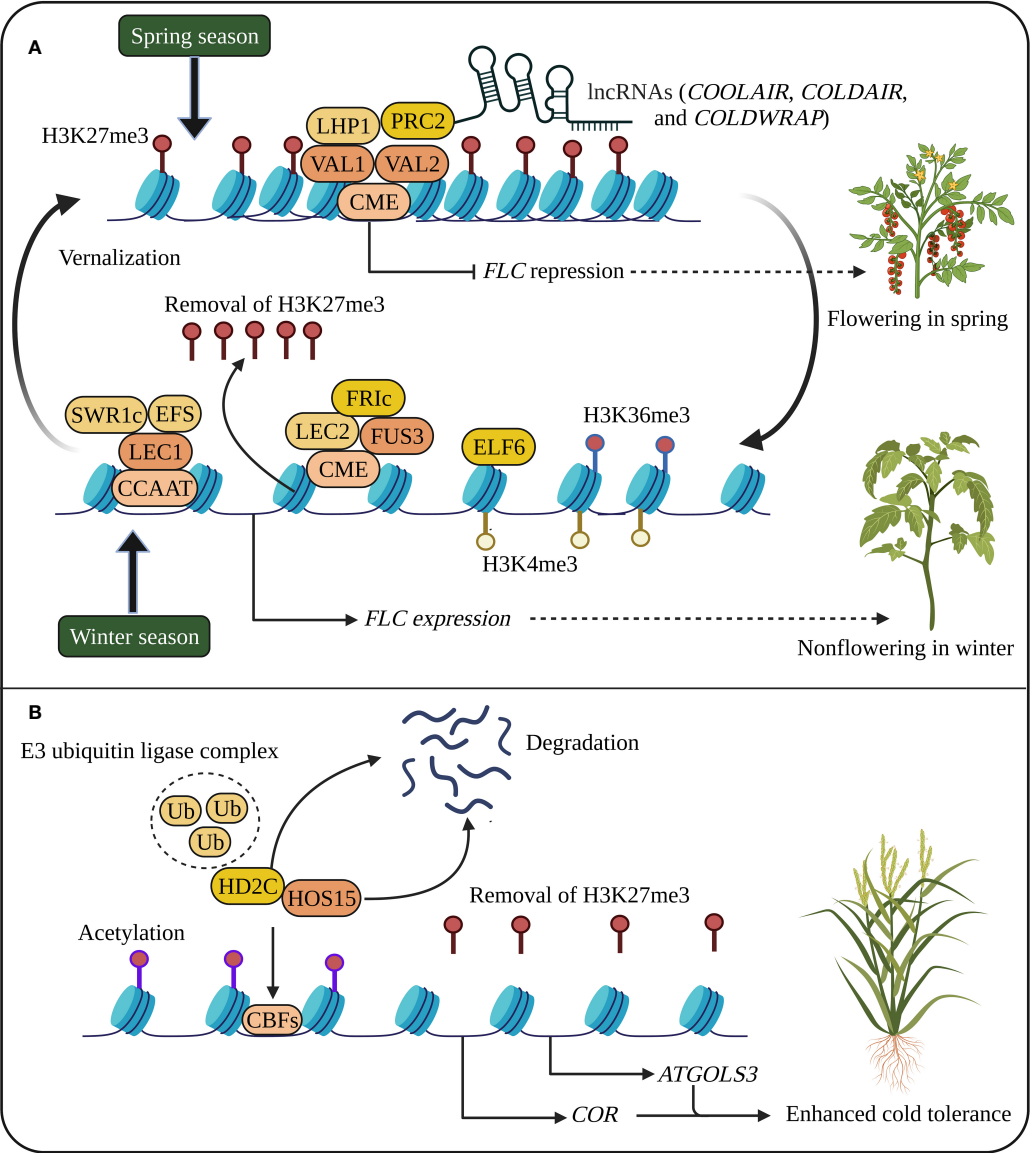
Cold stress memory in plants. **(A)** *Flowering Locus C* (*FLC*) expression suppresses flowering at high levels during winter. During vernalization, cold memory *cis*-acting element (CME), B3 transcription factors (TFs) such as *Viviparous1* (*VAL1* and *VAL2*), LIKE HETEROCHROMATIN PROTEIN 1 (LHP1), Polycomb repressive complex 2 (PRC2), and long noncoding RNAs (lncRNAs) silence *FLC* activation, and the spread of H3K27me3 throughout the *FLC* locus during cell division and DNA replication leads to *FLC* repression. In the next generation, epigenetic cold memory is reactivated during winter by the reactivation of *FLC* through TFs such as *Leafy Cotyledon 1* (*LEC1*), *LEC2* and *FUSCA3* (*FUS3*), *Early Flowering 6* (*ELF6*), *FRIGIDA* (*FRI*), *Early Flowering in short days* (*EFS*) and chromatin remodeling complex/PIE1 complex (SWR1c). **(B)** Cold stress memory regulates the expression of *Cold-regulated genes* (*CORs*) and *C-REPEAT-BINDING FACTORs* (*CBFs*) through histone methylation and acetylation. Under cold conditions, the CULLIN4 (CUL4)-based E3 ubiquitin ligase complex (Ub) degrades HIGH EXPRESSION OF OSMOTICALLY RESPONSIVE 15 (HOS15) and HISTONE DEACETYLASE 2C (HD2C), resulting in the activation of histone acetylation and *CORs* genes (Park et al., 2018). The reduced H3K27me3 also promotes the expression of *CORs* and *ATGOLS3*. The schematic representation was adapted from (Xie et al., 2021) by adding additional information and created using BioRender.com.

In rice, 19 HDAC genes have been identified (Hu et al., 2009), and the expression of *OsHDA714*, *OsSRT701*, and *OsSRT702* is modulated by cold stress (Fu et al., 2007). *AtHD2D* was induced in *Arabidopsis* subjected to cold stress, resulting in slowed seed germination and flowering but a slow increase in malondialdehyde content; thus, transgenic plants are capable of greater cold tolerance (Han et al., 2016). In *Arabidopsis*, the upregulation of HDA6 promotes freezing tolerance, and *hda6* mutants reduced cold tolerance by affecting hundreds of cold-responsive genes, divulging a key function of HDA6 in cold response (To et al., 2011). *High Expression of Osmotically Responsive gene 15* (*HOS15*) involved in histone deacetylation is also necessary for cold response. The *hos15* mutant increased the acetylation of histone H4, and HOS15 interacts with HD2C and *C-REPEAT-BINDING FACTOR* (*CBF*) transcription factors (**Figure 1**). The degradation *HOS15* and HD2C (a partner of *HOS15*), mediated by the CULLIN4 (CUL4)-based E3 ubiquitin ligase complex, triggers histone acetylation and *CORs* genes*, COR15A* and *COR47*, revealing the importance of HDACs and *HOS15*-mediated histone modifications in cold stress memory (Zhu et al., 2008; Park et al., 2018). However, further focused research on crop plants using advanced high-throughput sequencing technologies such as chromatin immunoprecipitation-sequencing (ChIP-seq) targeting histone modifications could lead to plant development with enhanced stress memory capable of tolerating cold stress.

## DNA methylation during cold stress in plants

Several studies reveal that DNA methylations, such as 5-methylcytosine (5mC) and N^6^-methyladenine (6mA), regulate transcriptional activities in response to cold stress (Zhang et al., 2018; Sicilia et al., 2020; Kidokoro et al., 2022; Verma et al., 2022). Compared with 6mA, a new epigenetic marker, 5mC is the most-studied modification under cold stress. Although 6mA is widely distributed across *Arabidopsis* and rice genomes and associated with gene expression and stress responses (Zhang et al., 2018; Liu and He, 2020), 6mA functions in stress memory remain elusive. Therefore, we limit this review to the discussion of 5mC. Plants are able to transmit cold-stress-induced DNA methylation changes within the generation or as transgenerational epigenetic memories, allowing plants to respond to cold stress a second time (Sharma et al., 2022).

DNA methylation variations in response to cold acclimation were detected in *Chorispora bungeana* (Song et al., 2015), *Populus simonii* (Song et al., 2016) and *Phyllostachys edulis* (Ding et al., 2022). Cold acclimation induces both DNA methylation and demethylation; and demethylation regulates freezing tolerance in *Arabidopsis*. In cucumbers, cold stress impacts RNA-directed DNA methylation (RdDM), resulting in demethylation. However, the lowered activity of *MSH1* and *RNA-DIRECTED DNA METHYLATION 4* (*RDM4*), a key player in RdDM pathway, reduced cold response in *Arabidopsis*. *RDM4* modulates the expression of *CBF* TFs, which regulates cold stress memory (Liu and He, 2020; Xie et al., 2021). Cold stress reduced 5mC in vernalized plants and increased the expression *CASEIN KINASE II A-SUBUNIT* (*CKA2*) and *B-SUBUNIT* (*CKB4*), which elevated CK2 activity, resulting in the reduction of the clock gene *CIRCADIAN CLOCK ASSOCIATED 1* (*CCA1*), an important player in photoperiod perception (Duan et al., 2017). Vernalization-induced DNA demethylation is not conserved in plants, and vernalization also influences non-CG methylation that can be maintained *via* mitosis (Liu and He, 2020).

5mC modulates ICE1-CBF-COR pathway associated with cold tolerance. In *Hevea brasiliensis*, the expression of *HbICE1* and *HbCBF2* was associated with DNA demethylation and cold tolerance (Tang et al., 2018). In *Arabidopsis*, the 5mC variation in *INTERACTOR OF LITTLE ELONGATION COMPLEX ELL SUBUNIT 1* (*ICE1*) and *ALLANTOINASE (ALN)* regulated freezing tolerance and seed dormancy and stimulated non-canonical RDR6 and ARGONAUTE 6 (AGO6)-dependent RdDM pathway. DNA hypermethylation of *AtICE1* resulted in the suppression of the genes involved in CBF pathway (Iwasaki et al., 2019; Xie et al., 2019). In *Brassica rapa*, cold-induced DNA demethylation promoted the expression of *MITOCHONDRIAL MALATE DEHYDROGENASE* (*BramMDH1*), whose overexpression increased heat tolerance in *Arabidopsis* but not in *Brassica rapa.* This indicates that DNA demethylation alone is not sufficient to increase heat tolerance in *Brassica rapa* but is sufficient to increase cold tolerance (Liu et al., 2017a). In *Rosa hybrida*, cold-induced DNA methylation (CHH context) reduced the expression of *RhAG*, whose improved suppression increased cold-induced petal number (Ma et al., 2015). In addition, reduced DNA and histone methylations (H3K9me2) and increased histone acetylation (H3K9ac) in heterochromatic regions regulate cold tolerance, suggesting that changes in both DNA and histones are necessary for cold acclimation (Kim et al., 2015). Like 5mC, the 6mA level is also reduced in response to cold stress (Zhang et al., 2018), but how both 5mC and 6mA functions connected in freezing tolerance remain largely obscure. Therefore, the characterization of cold stress-responsive epigenetic modifications is crucial for developing plants with enhanced cold stress memory.

## Genes involved in cold acclimation in plants

Low temperatures cause membrane stiffening leading to calcium influx into cells and triggering signalling cascades regulated by multiple genes that lead to cold acclimation, suggesting that cold stress is sensed by plant cells through changes in the fluid state of the plasma membrane (Ma et al., 2015; Ding et al., 2019). Cold stress induces the expression of many transcription factors, such as *CBF*. The cold-responsive CBF signalling pathway is one of the known characterised mechanisms for transcriptional cold memory (Hwarari et al., 2022). In *Arabidopsis*, CBF proteins bind to the *cis*-acting element DRE/CRT present in the promoter regions of the cold-responsive genes such as *COR* genes and activate their expression (**Figure 1**) (Adhikari et al., 2022). Cold stress rapidly induces *CBF1, CBF2* and *CBF3* genes along with *COR* gene expression. The overexpression of *CBFs* genes in plants increased cold tolerance and plant growth and development (late flowering) (Guo et al., 2018).

Depending on cold-induced epigenetic changes, the *CBF* genes are either positively or negatively regulated by other TFs or genes. For example, the mutant *cbf2* in *Arabidopsis* increased freezing tolerance and cold acclimation, but negatively regulated *CBF1* and *CBF3* (Novillo et al., 2004). COLD-RESPONSIVE PROTEIN KINASE 1 (CRPK1) is a cold-activated plasma membrane-localized protein and a negative regulator of CBF pathway. CRPK1 phosphorylates 14-3-3 proteins which interact with and destabilize CBF proteins in the nucleus, thus playing a negative role in cold acclimation (Liu et al., 2017c). Whereas the expression of *CBFs* is positively regulated by *ICE1* and *ICE2* (bHLH TFs), thereby maintaining the activation of *CBF/COR* genes (Guo et al., 2018). To degrade *ICE1* and to suppress the *ICE1*-mediated expression of *CBF/COR* genes, *ICE1* is ubiquitinated by *HIGH EXPRESSION OF OSMOTICALLY RESPONSIVE GENES1* (*HOS1*) under cold stress. However, SIZ1 and SIZ2 modulate *ICE1* SUMOylation to suppress *ICE1* degradation, thus activating *CBF/COR* genes to increase cold memory and tolerance (Guo et al., 2018). The HOS15-HD2C complex promotes hypoacetylation of *COR* chromatin to inhibit *COR* genes, but the CUL4-based E3 ubiquitin ligase complex degrades HD2C and induces hyperacetylation of *COR* genes, which enhances the binding activity of CBFs to the promoters (Park et al., 2018). Therefore, the *CBF*-dependent gene expression through epigenetic regulation is a significant component of cold memory and acclimation in plants. However, in *Arabidopsis*, the transcriptome analysis showed that only 12% of the cold-responsive genes were regulated by *CBF* (CBF regulon/CBF-targeted genes), and no less than, 28% of the cold-responsive genes were not regulated by *CBF*, indicating that cold-responsive genes and cold memory genes are regulated by different TFs (Fowler and Thomashow, 2002).

*NON-EXPRESSER OF PATHOGENESIS-RELATED GENES 1* (*NPR1*) gene is known to be the master regulator of plant pathogen response. *NPR1* is epigenetically regulated; indeed, the histone methyltransferase SET DOMAIN GROUP 8 (SDG8) binds it and induces upon H3K36 methylation (H3K36me3) its transcription upon pathogen infection (Zhang et al., 2020). However, recent genetic and molecular evidence has shown that *NPR1* plays an essential role in cold acclimation by independently regulating cold-induced gene expression of salicylic acid and TGA TFs (Olate et al., 2018). Also, the fact that *NPR1* might serve as a central hub integrating cold and pathogen signalling for a better adaptation is very interesting. Therefore, it seems important to insist that stress responses (biotic/abiotic) are intimately connected. In addition, understanding different signalling pathways in cold stress memory is required to improve cold stress response.

## Regulation of heat stress in plants

Heat stress can cause cellular damage and cell death and produces an excessive amount of reactive oxygen species (ROS) and oxidative stress, resulting in morphological, physiological and biochemical changes, or in leaf shedding, flower and fruit abortion or even plant death (Hasanuzzaman et al., 2013). Heat-induced regulated cell death (RCD) such as apoptosis, necrosis and ferroptosis pathways selectively removes certain cells in specific tissues to maintain homeostasis under heat stress (Distéfano et al., 2021). Plant response to heat stress is a complex process that differs among species depending on the temperature range (28-48°C) and is regulated by different mechanisms such as hormone signalling, transcriptional process and epigenetic changes. The histone proteins H1, H2A, H2B and H3 with the exception of H4 have specialized histone variants that reprogram the gene expression responsible for heat acclimation and heat stress memory. Several key genes and networks regulating the heat response have been identified, and heat stress memory is associated with epigenetic changes (Li et al., 2021; Malik and Zhao, 2022). In contrast to hormonal regulation underlying heat stress at the transcriptional level, epigenetic regulation under stress in plants remains elusive, suggesting that identifying key mechanisms is important. The next sections will briefly discuss the epigenetic mechanisms and genes involved in heat stress response and memory.

## Histone modifications during heat stress

Heat acclimation depends on a specific pattern of gene expression regulated by histone modifications, dynamic in response to heat stress and associated with gene transcription (Pecinka et al., 2010; Bhadouriya et al., 2021). Some histone modifications change rapidly under stress conditions, while others adapt gradually to the response (Kim et al., 2015). Histone H2A.Z, a variant of H2A comprising H2A, H2A.Bbd, H2A.X and H2A.Z variants, is involved in temperature sensing globally through their nucleosome occupancy. In *Arabidopsis*, the insertion of the histone variant H2A.Z into nucleosomes is regulated by a subunit of the chromatin remodeling complex (SWR1), replacing H2A with H2A.Z in an ATP-dependent manner. *ACTIN-RELATED PROTEIN6* (*ARP6*) gene encodes SWR1, suggesting that *ARP6* is required for histone modification and mediates temperature response genes (Kumar and Wigge, 2010). In *Brachypodium*, the *H2A.Z* mutant decreased heat acclimation (Boden et al., 2013). Histone acetylation is necessary for H2A.Z deposition. This suggests that other histone variants and H2A.Z-containing nucleosomes modulate transcription in a temperature-dependent manner (Kim et al., 2015). Heat stress increases histone acetylation (H3K9ac and H4K5ac) in different tissues of maize and either does not alter or decreases histone methylation, divulging a key role of histone acetylation and methylation in chromatin decondensation (Wang et al., 2015). In *Arabidopsis*, the chromatin protein BRUSHY1 (BRU1) involved in chromatin organization facilitates the epigenetic inheritance of chromatin states and maintains the transcriptional induction of heat stress memory genes (Brzezinka et al., 2019).

The down-regulation of histone acetyltransferase GCN5 reduces heat tolerance. Heat tolerance of *Arabidopsis* depends on the expression of stress-related genes mediated by GCN5. Induction or overexpression of stress-related genes in the *gcn5* mutant increased stress memory and restored stress tolerance (Hu et al., 2019). HDACs such as HD2C, HDA6, HDA9, HDA15, and HDA19 regulate stress responses either negatively or positively. For example, in *Arabidopsis*, the interaction of HDA9 with POWERDRESS (PWR), a SANT-domain containing protein, increases heat tolerance (Tasset et al., 2018). In contrast, HDA15 decreases heat tolerance while interacting with TF, long Hypocotyl in Far Red1 (Shen et al., 2019).

**Figure 2:**
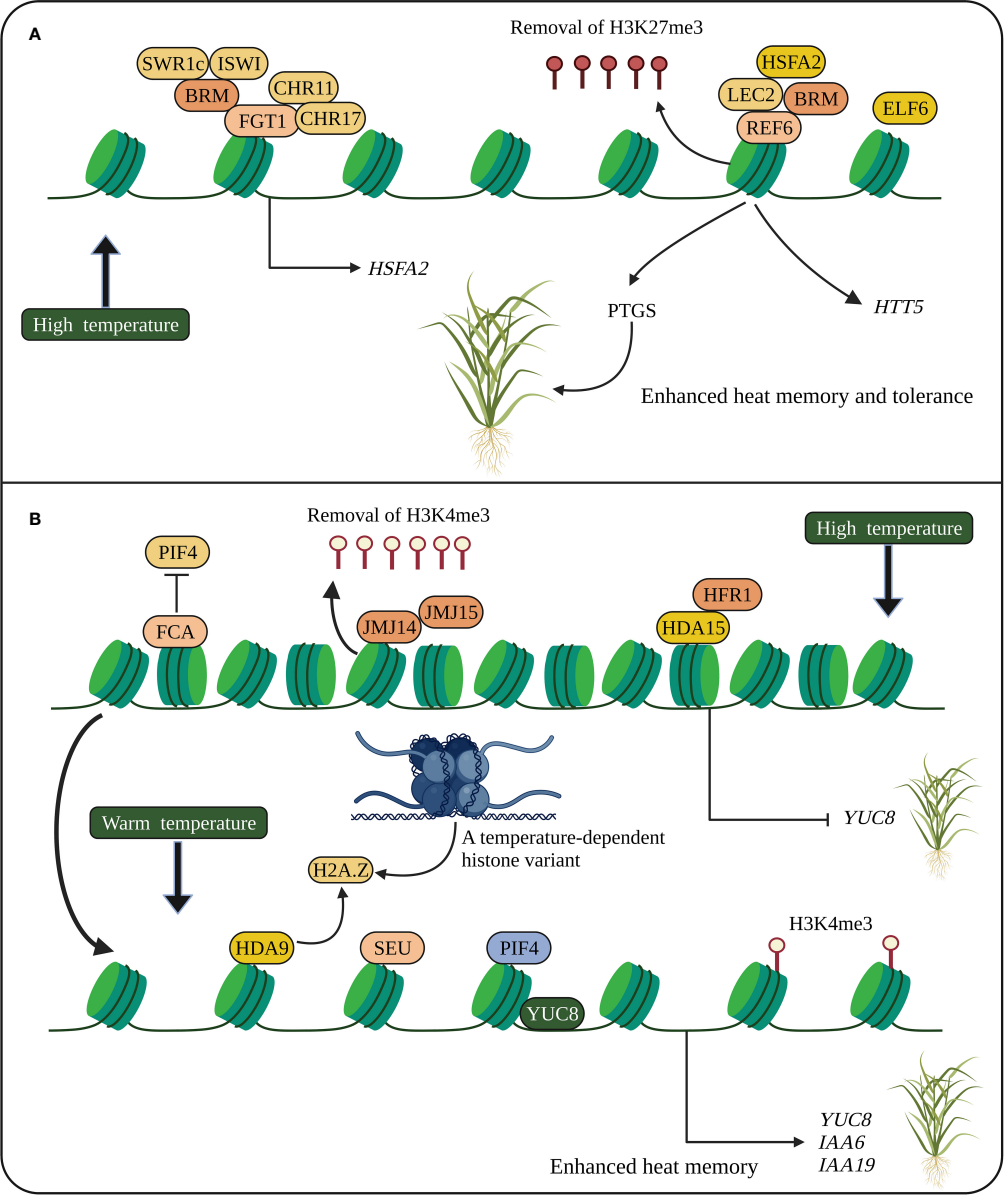
Heat stress memory in plants. **(A)** Heat memory is regulated by HSFA2-REF6-PTGS and FGT1. HSFA2 and REF6 are essential for heat memory and trigger the SGS3-interacting protein, which degrades SGS3 and inhibits PTGS. REF6 removes H3K27me3, and FGT1 and the chromatin remodelers such as BRM, CHR11 and CHR17 modulate nucleosome occupancy at the loci of heat memory genes. **(B)** Stress memory (thermomorphogenesis) is also regulated by PIF4. Epigenetic modifications and chromatin remodelers activate PIF4 to promote stress memory genes. A temperature-dependent histone variant (H2A.Z) modulating gene network is associated with YUC8 to promote PIF4 activation. HSFA2, heat shock transcription factor; *REF6*, *RELATIVE OF EARLY FLOWERING 6*; PTGS, posttranscriptional gene silencing; *SGS3*, *SUPPRESSOR OF GENE SILENCING 3*; *FGT1*, *FORGETTER1*; BRM, BRAHMA; CHR11 and CHR17, CHROMATIN-REMODELING PROTEIN 11 and 17, respectively; JMJC14 and JMJC15, Jumonji C domain-containing protein 14 and 15, respectively; *PIF4*, *PHYTOCHROME INTERACTING FACTOR 4*; *FCA*, *FLOWERING CONTROL LOCUS A*; *SEU*, *SEUSS*; *YUC8*, *YUCCC8*; *IAA*, *INDOLE-3-ACETIC ACID INDUCIBLE*. The schematic representation was adapted from (Xie et al., 2021) by adding additional information and created using BioRender.com.

Histone methylations regulate heat stress responses and memory. In *Arabidopsis*, H3K4me3 correlates with active gene expression, whereas the correlation of H3K4me2 with active transcription is negative in rice. A decrease in H3K4me2/H3K4me3 levels misregulated many genes related to plant development. Expression of several memory genes such as *HSP18.2*, *HSP21*, and *HSP22.0* correlates with H3K4me3 and H3K4me2 at their respective target loci, and higher accumulation of these modifications is associated with hyper induction of memory genes under continuous heat stress (Lämke et al., 2016; Bäurle, 2018; Liu et al., 2019). Like the regulation of cold acclimation by reduced H3K27me3, heat tolerance is also regulated by reduced H3K27me3 at *HSP17.6C* and *HSP22* genes mediated by JMJC histone demethylases, thus increasing heat stress memory in *Arabidopsis*. In addition, the removal of H3K27me3 by JMJC (**Figure 2**) is to maintain the accumulation of H3K4me3 at *HSP21* gene during heat response, suggesting that the balance between H3K27me3 and H3K4me3 levels is required for stress memory (Yamaguchi and Ito, 2021). Reduced H3K9me2 attenuates heat responses and is an important component in the regulation of *Fertilization-Independent Endosperm1* (*OsFIE1*), a member of PRC2, in *Arabidopsis* seed development under moderate heat stress (Folsom et al., 2014).

Histone SUMOylation contributes to the transcriptional regulation mediated by histone modifier HDACs and has conserved functions in heat acclimation. In *Arabidopsis*, heat stress decreased histone SUMOylation (H2B) and increased GCN5, and a variety of histone modifiers are involved in the regulation of SUMOylation (Kim et al., 2015). However, the modifications and their association with stress memory genes remain elusive and require considerable attention to investigate their functions in stress memory.

## DNA methylation during heat stress in plants

Although studies show that DNA methylation (either hypomethylation or hypermethylation) regulates transcriptional activities in response to heat stress (Ding et al., 2022; Perrella et al., 2022; Sun et al., 2022), the relationship between DNA methylation and heat stress memory is still largely unclear. DNA methylation mediated by the RdDM pathway is known to regulate several heat stress-responsive genes, and this pathway is required for basal heat tolerance and the transition of transgenerational memory in *Arabidopsis*. Furthermore, *Arabidopsis* mutants with epigenetic defects showed that the transcriptional heat response depends on the RdDM pathway and HDA6 (Popova et al., 2013; Yang et al., 2020). In *Brassica napus*, DNA methylation changes between heat-tolerant and heat-sensitive genotypes, and the heat-sensitive genotypes displayed hypermethylation, suggesting that the level of DNA methylation is dynamic in response to heat stress (Gao et al., 2014). Heat stress induced active demethylation in *Arabidopsis*, especially after returning to normal temperature, and differentially methylated genes are associated with heat response (Korotko et al., 2021). Demethylation facilitates intergenerational stress memory and differentially methylated regions (DMRs) enriched with transposons were linked with the stress memory dependent on DNA methylation (Wibowo et al., 2016).

Heat-accelerated DNA methylation also regulates transposon silencing or activation to ameliorate stress memory. For instance, in *Arabidopsis* under heat stress, CHROMOMETHYLASE3 (CMT3), a DNA methyltransferase, activated retrotransposon *ONSEN* that is capable of binding heat shock (HS) TFs involved in transcriptional memory and is transcriptionally activated and increased their copies, which are involved in transgenerational stress memory. The *cmt3* mutants increased methylation at *ONSEN*, but CMT2-bound *ONSEN* chromatin in *cmt3* mutants accumulated H3K9me2, suggesting a collective role of transposon functions connected with DNA and histone modifications under stress conditions (Ito et al., 2011; Cavrak et al., 2014). In addition, retrotransposons maintained by RdDM suppress chromatin modifications and perform a role in stress memory. The transpositions of retrotransposon occur more frequently in the heat-stressed progeny of RdDM mutants, implying that the RdDM machinery plays an important role in retrotransposon silencing and prevents the transgenerational memory of retrotransposons in *Arabidopsis* (Matsunaga et al., 2012; Sharma et al., 2022). However, the HS *cis*-acting element in the retrotransposon’s promoter region can bind HS TFs and enable the transcription under heat stress conditions to enhance stress memory (Cavrak et al., 2014).

In maize, heat-induced demethylation is associated with the spliceosome pathway, suggesting that DNA demethylation regulates heat responses *via* RNA splicing (Qian et al., 2019). The interaction of spliceosome complex proteins with nuclear cyclophilins (CYPs) associated with spliceosomal components is required for heat acclimation in *Arabidopsis* (Jo et al., 2022). In addition, heat-induced genome-wide hypomethylation disrupted the metabolic pathways related to sugars and reactive oxygen species (ROS) (Liu and He, 2020). In addition, heat stress inhibited cell division in the tobacco cell line (BY-2 cells) and arrested cells in the pre-mitotic phase (somatic cells) by increasing the expression of *CycD3-1* and decreasing the transcripts of *NtCycA13*, *NtCyc29* and *CDKB1-1*. This was due to the altered expression of *CycD3-1* and *Nt-EXPA5* in conjunction with the methylation status of their promoters, suggesting that DNA methylation regulates cell cycle progression to control mitosis involved in somatic stress memory (Centomani et al., 2015).

Heat-induced DNA methylation in *Arabidopsis* affected two genes *At3g50770* (calmodulin-like 41(CML41) and *At5g43260* (Chaperon-like protein) containing transposon insertions in their promoters, and *At3g50770* expression is correlated with its promoter methylation. Heat-induced *NUCLEAR RNA POLYMERASE D1A* (NRPD1A) and *NRPD1B* upregulate *CML41* under heat stress. This suggests that the RNA polymerases PolIV and PolV (NRPD1 and NRPE1 respectively), main players in DNA methylation, may regulate transcripts not only through RdDM but also through other mechanisms (Naydenov et al., 2015). *Arabidopsis* mutants lacking DNA methyltransferase, such as *domains rearranged methylase1* (*drm1*), *drm2* and *cmt3*, showed less distinct-heat response; however, the prolonged-heat stress downregulated *METHYLASE1* (*MET1*) and *CMT3*, confirming that DNA methylation is dynamic under heat stress (Naydenov et al., 2015). However, the role of DNA methylation and its interaction with stress memory is poorly understood and requires considerable attention to investigate the role of DNA methylation in heat stress epigenetic memory.

## Genes involved in heat tolerance

Several candidate genes and TFs associated with heat tolerance and stress memory have been found in many plant species. As molecular chaperones, heat shock proteins (HSPs) encoded by HSP family genes protect cells from heat stress through various strategies, including targeted protein localization. HSPs are classified into different families such as HSP10, HSP20, HSP40, HSP60, HSP70, HSP90 and HSP100 (Hasanuzzaman et al., 2013). HSPs are regulated by heat shock TFs (HSFs) interacting with heat-induced TFs and enzymes (Samakovli et al., 2020; Bourgine and Guihur, 2021). The heat shock response (HSR) and heat-responsive cyclic nucleotide-gated ion channels (CNGCs) trigger heat-induced gene expression to accumulate HSPs (Guihur et al., 2022).

Genes such as *HSP17.6C*, *HSP21* and *HSP22*, members of the small HSPs (sHSPs), are key factors of heat stress memory and associated with histone methylation (H3K4me3 and H3K27me3). *JMJ* genes balance H3K4me3 and H3K27me3 at *HSP21* during heat acclimation and activate *HSP* genes when sensing heat stress, whereas *jmj* mutants do not maintain heat memory (Yamaguchi and Ito, 2021; Yamaguchi et al., 2021). In *Arabidopsis*, the enzyme Filamentation Temperature-Sensitive H6 (FtsH6) degrades or resets HSP21 abundance during heat stress recovery. *HSFA2*, a positive regulator of stress memory and a member of the family of 21 heat stress TFs, activates *FTSH6* and improves stress memory in *ftsh6* mutants compared with wild-type plants (Sedaghatmehr et al., 2022).

The overexpression of *HSFA2* in *Arabidopsis* activates downstream targets *via* heat stress elements (HSEs) and induces the expression of *HSP21*, *HSP22*, *HSP18.2*, and *ASCORBATE PEROXIDASE 2* in response to heat stimuli (Yamaguchi, 2022). *JMJ*-and *HSFA2*-mediated histone modifications occur at the same *HSP22* locus. The mutants such as *hsp21, hsp22, hsp17.6c*, *heat-stress-associated 32* (*hsa32*) and *hsfa2* decreased heat acclimation, whereas the overexpression of these genes improved heat acclimation and memory (Yamaguchi, 2022), intimating that heat memory genes are *JMJ* and *HSFA2* dependent. Unlike the activation of *HSFA2*, the repression of *SQUAMOSA promoter-binding protein-like* (*SPL*) TF triggers heat memory genes (Yamaguchi, 2022). *HSFA1* and *HSFA2* TFs circuitously regulate *AGAMOUS LIKE 16* (*AGL16*), encoding a MADS-box TF and acting as a post-transcriptional memory factor (Xie et al., 2021). These results elucidate how heat memory ensures heat acclimation.

*HEAT-INDUCED TAS1 TARGET* (*HTT*) genes confer heat tolerance through the action of HSPs such as Hsp70-14, Hsp40, NUCLEAR FACTOR Y and SUBUNIT C2. *HSFA1a* TF binds directly to the HSE in the *HTT* promoter and can induce heat tolerance in *Arabidopsis* (Li et al., 2014). *FORGETTER1* (*FGT1*), encoding a plant homeodomain (PHD) finger protein, physically interacts with SWI2/SNF2 chromatin remodelers (BRAHMA (BRM) and CHROMATIN-REMODELING PROTEIN 11 (CHR11) and CHR17) to enhance heat stress memory by promoting heat-responsive genes. *FGT1* regulates nucleosome dynamics at the gene loci of *HSP22*, *HSP18.2*, *HSP21* and *HSA32* after initial heat stress and mediates stress memory (Brzezinka et al., 2016). The *fgt1* mutant reduced *HSA32* expression, thereby impairing heat acclimation. The mutants *brm and chr11 chr17* also showed heat memory defects (Yamaguchi, 2022). *FGT2*, encoding a TYPE-2C PROTEIN PHOSPHATASE (PP2C), interacts with PHOSPHOLIPASE D α2 (PLDα2), which activates lipid composition and stress memory. Like the *fgt1* mutant, the *fgt2* mutant also exhibited an increased heat memory defect, indicating that *FGT1* and *FGT2* are essential for heat memory (Xie et al., 2021).

Heat-induced post-transcriptional gene silencing (PTGS) shows transgenerational stress memory, and the *SUPPRESSOR OF GENE SILENCING 3* (*SGS3*) is required for PTGS. *HSFA2* and *RELATIVE OF EARLY FLOWERING 6* (*REF6*) regulate heat memory transmission. HSFA2-REF6 triggers SGS3-interacting protein, which inhibits PTGS by degrading SGS3 (**Figure 2**) (Xie et al., 2021). Small RNA-regulated *AGO1* involved in PTGS is also required for heat stress-induced transgenerational inheritance in *Brassica rapa* (Bilichak et al., 2015). Stress memory (thermomorphogenesis) is also regulated by the bHLH TF, *PHYTOCHROME INTERACTING FACTOR 4* (*PIF4*), induced at warm temperatures. The chromatin remodeler and epigenetically activated PIF4 bind directly to the *YUCCC8* (*YUC8*) promoter to promote stress memory at warm temperatures. The RNA-binding protein *FLOWERING CONTROL LOCUS A* (*FCA*) removes the occupancy of PIF4 from *YUC8* by interacting with a histone demethylase to mediate stress memory (**Figure 2**) (Xie et al., 2021). Altogether suggests that *HSPs*, *HSFs* and *SGS3* are key regulators of heat stress memory. However, how HSFs and JMJs interact with HSPs and regulate histone modifications remains to be elucidated.

## Stress-induced epigenetic memory and its heritability

Several studies show that plants not only store their stress experiences but also retain them over multiple generations (Latzel et al., 2016; Liu et al., 2022b). In general, stress-induced epigenetic modifications return to baseline levels after the stress ends. However, some modifications remain stable and can be inherited as epigenetic stress memory over multiple generations through mitotic and meiotic cell divisions, while other modifications are reactivated during sexual reproduction or embryogenesis (D’urso and Brickner, 2014; Tricker, 2015). Epigenetic memory, without alteration of nucleotide sequences, requires distinct chromatin changes and impacts gene activation and related mechanisms on different time scales and under different environmental stresses. Cellular, transcriptional, and transgenerational memory are the three types of epigenetic memory that are dynamic and mitotically or meiotically heritable and triggered by an antecedent stress exposure. Cellular memory (somatic) is a mitotically heritable transcriptional state established during primary development with tissue-specific TFs and transcriptional programs. Transcriptional memory is also mitotically heritable changes for several generations in response to previous stress experiences. In contrast, transgenerational memory is meiotically heritable changes and can impact the offspring’s behaviour (D’urso and Brickner, 2014).

**Figure 3:**
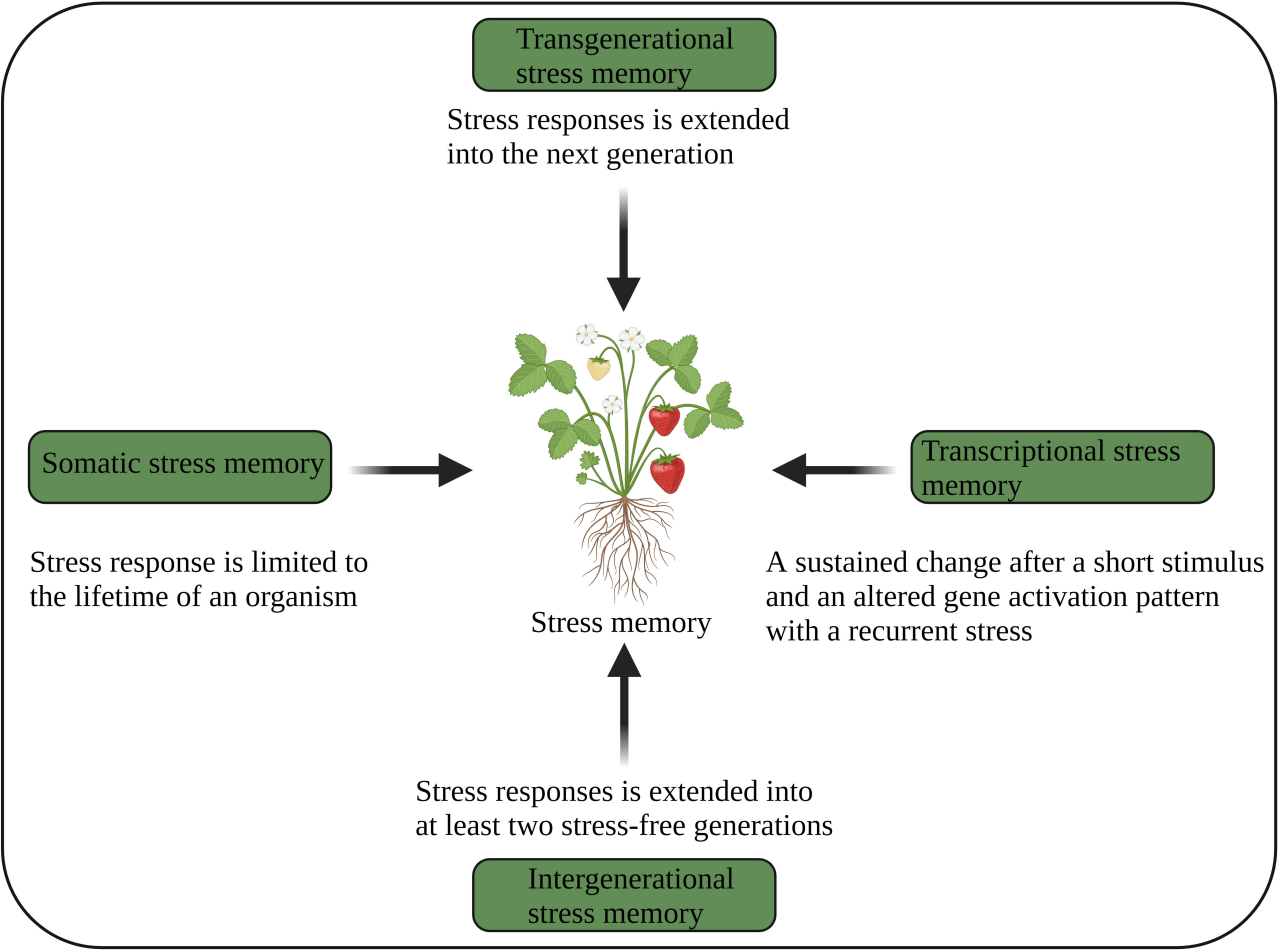
Different aspects of plant stress memory (Oberkofler et al., 2021). The image was created using BioRender.com.

Plants use transcriptional, somatic, intergenerational and transgenerational stress memory (**Figure 3**), and several epigenetic mechanisms regulating those memories have been identified (Bäurle and Trindade, 2020; Sharma et al., 2022). Somatic stress memory lasts only for a specific period of time within a generation (a few days or weeks). For example, vernalization memory is maintained for a few weeks by H3K27me3 of the *FLC* gene (Yang et al., 2017). PRC1 and PRC2 are required to keep somatic memory genes active by mediating histone methylation (H3K27me), which is a repressive bookmark during mitosis and is obligatory for the maintenance and inheritance of active chromatin states. This suggests that somatic memory necessitates both chromatin repressive and modifying complexes (D’urso and Brickner, 2014).

Transgenerational memory continues into the next stress-free generation, where epigenetic changes are independent (Shanker et al., 2020). The genome-wide distribution of DNA methylation and regulatory mechanisms involving chromatin modifications play an important role in the transgenerational inheritance of stress memory (Ou et al., 2012; Tricker, 2015; Cong et al., 2019). Intergenerational memory persists for at least two stress-free generations (Lämke and Bäurle, 2017; Oberkofler et al., 2021). Nevertheless, intergenerational memory is reversible after a stress-free generation, suggesting that this memory is stress-dependent. This memory is mainly maternally inherited. The DEMETER (DME) (DNA demethylase) suppresses paternal inheritance, but paternal inheritance is restored in *dme* mutants. The RdDM-mediated demethylation regulates intergenerational memory (Wibowo et al., 2016). However, how intergenerational and transgenerational memories are inherited remains unclear.

Histone H3K4me2 and H3K4me3 regulate acquired transcriptional memory in primed plants (Jaskiewicz et al., 2011), and the hypermethylation of these marks maintains transcriptional memory during heat stress (Lämke et al., 2016). Besides this, transcriptional memory also requires histone variant H2A.Z, and the mutants lacking this variant did not retain memory (D’urso and Brickner, 2014). Not all genes are stress-induced memory genes. Thus, memory genes have been classified into two types: 1, genes that show persistent activation or suppression after exposure to stress, and 2, genes that show altered response to recurrent stress compared to the response of naive plants (Bäurle, 2018). However, because plants use both male and female specific germline mechanisms (Ono and Kinoshita, 2021), stress-induced epigenetic memory is still unclear, although there are several examples of priming of plant defences and evidence of induced genetic rearrangements. Therefore, further studies are needed to clarify the involvement and persistence of epigenetic memory.

## Emerging technologies to analyse stress-induced epigenetic memory

Although high-throughput sequencing has facilitated genome assembly and gene annotation, the accurate annotation and assembly remain complex due to pseudogenes and duplicate copies of transposons. This prevents the application of stress memory in crop improvement. By using artificial intelligence to find predictive patterns in data and perform specific tasks based on a given dataset, machine learning could improve the annotations of transcriptomics, epigenomics and proteomics and accurately classify differentially expressed genes, proteins, pseudogenes and transposon copies (Libbrecht and Noble, 2015; Sartor et al., 2019). Machine learning can also highly accurately detect tissue-specific methylation profiles and gene expression in crop plants (N’diaye et al., 2020). Deep learning, a subset of machine learning, is also an emerging tool for genomics and its functional annotations and can uncover stress-induced interactions between chromosomes and genes, including lncRNA-miRNA interactions between different regulatory elements (Xu et al., 2022). In addition, deep Learning contributes to a better understanding of the topology of the genetic network and phenotype changes, as well as to the automation of genome prediction for various epigenetic changes in plants (Kautsar et al., 2017; Gazestani and Lewis, 2019; Wang et al., 2021a).

Oxford Nanopore sequencing, a third-generation approach, can sequence DNA and RNA without chemical labelling and PCR amplification of the sample and supports profiling of genome, epigenome, transcriptome and epitranscriptome with a single assay with 98.3% accuracy (Wang et al., 2021b; Wan et al., 2022). Machine learning can detect DNA modifications by analysing ion current signals from direct DNA sequencing with nanopores (Wan et al., 2022). In addition, single-cell sequencing (Tang et al., 2019) and the assay for transposase-accessible chromatin using sequencing (ATAC-seq) (Ji et al., 2020; Grandi et al., 2022) await applications to investigate how DNA and histone modifications interact with gene expression in individual cells during stress memory stimulation. High-throughput phenotyping has emerged as a new perspective for non-destructive field-based phenotyping (Jangra et al., 2021). These technologies offer new opportunities to study stress-induced epigenetic memory, but these methods are insufficient for memory development because epigenetic changes have low specificity. Further improvements in machine learning and Oxford Nanopore sequencing will lead to a better understanding of epigenetic regulation and stress memory. A trained deep and machine learning algorithm with high-throughput sequencing and phenotyping can be used in plant phenology. The method could also be used to identify and classify plant stress and quantify stress memory expression for future plant stress and further development.

## Future perspective to increase epigenetic stress memory

1. A global database of chromatin changes (e.g., plant chromatin state database (PCSD, http://systemsbiology.cau.edu.cn/chromstates) (Liu et al., 2017b)) in plants grown at low and high temperatures is essential for a better understanding of epigenetic stress memory and may serve as an advanced research option to improve stress memory.

2. Molecular characterization of epigenetic stress memory is essential to link plant stress responses to phenotypic traits at all levels. The epigenetic regulation of certain stress-induced metabolites, such as volatile carbons, needs to be elucidated because they often function as interspecies communication molecules.

3. Some plant species are known to possess certain unique properties for stress tolerance and are more important than any other organism. Therefore, future research should focus on the functions of HSPs that are not yet elucidated in these plants, and extensive efforts are needed to identify specific epigenetic determinants that protect plants from the deleterious effects of cold and heat stress.

4. Practical implementation of epigenetic modifications is expensive compared to conventional plant breeding, which takes 5-10 years to develop new varieties. Therefore, a global initiative to optimise the cost-benefit ratio is needed to utilise epigenetic modification and its memory in plant breeding.

5. The use of epimutations or epialleles is a highly recommended technique to increase epigenetic stress memory, and technological advances in high-throughput sequencing and phenotyping could significantly alter this effective strategy.

6. Since epigenetic changes can be inherited by offspring as stress memory, targeted studies should be conducted to decipher the epigenetic codes of plant responses under stress conditions to further improve stress memory. Further development of high-throughput chromatin profiling could lead to significant progress in deciphering epigenetic changes and stress memory.

7. Determining the precise relationship between epigenetic modifications such as DNA methylation and histone modifications associated with epigenetic traits could lead to the development of cold and heat stress memory in crops. However, we are still trying to understand the interplay between specific epigenetic changes and epigenetic traits. Therefore, further studies could relate DNA methylation and histone modification to epigenetic traits and memory.

8. The heritability of stress-induced histone and DNA modifications needs further investigation, as these modifications are inherently dynamic and require epigenetic protein complexes to function properly.

9. The advancement of deep learning and machine learning with high-throughput sequencing could advance epigenetic research and thus the understanding of stress memory. High-throughput epigenetic studies on the mechanisms involved in the transmission of epigenetic memory are also needed to understand epigenetic memory better.

10. Creating big-data databases (e.g., quantitative PTMs, (http://qptmplants.omicsbio.info), a valuable resource for plant PTMs (Xue et al., 2022)) for epigenomics and the interpretation of multidimensional data are important to achieve a higher level of genetic gain. In addition, exploring epigenetic regulation using machine learning and big data tools could lead to a centralised infrastructure and data consortium for a better understanding of epigenetic memory that will greatly improve epigenetic stress memory.

11. Plant priming, i.e., exposure to non-lethal stress maintained over a period of time, is an adaptive method to enhance epigenetic memory, enabling them to respond robustly after second stress exposure (Ling et al., 2018). Therefore, artificially induced independent epigenetic changes through plant priming could lead to transgenerational inheritance (Bilichak and Kovalchuk, 2016).

## Conclusion and future opportunities

Plants require optimal temperature for ideal growth, and the stress response in higher plants is a complicated process that differs from species to species. The plant stress response is regulated by thousands of genes in conjunction with epigenetic mechanisms that are dynamic depending on different stress conditions. The epigenetic mechanisms, inherited as epigenetic stress memory through mitotic and meiotic cell divisions, are well understood in *Arabidopsis*, but the mechanisms in crop plants are still unknown. Epigenetic stress memory fine-tunes the relationship between DNA and histone modifications and regulates the transition of epigenetic changes. Stress-induced H3K9me2 and H3K27me3, associated with DNA methylation and chromatin structures, regulate stress memory and play important roles in memory development. However, it is still unclear how epigenetic memories are inherited and how they are maintained. Enzymes such as writers, readers and erasers of epigenetic changes involved are still mysterious in stress memory; thus, it is essential to identify novel enzymes and their functions. Therefore, discovering the exact mechanism regulating the transmission of epigenetic memory will lead to an improvement in stress memory.

**Figure 4:**
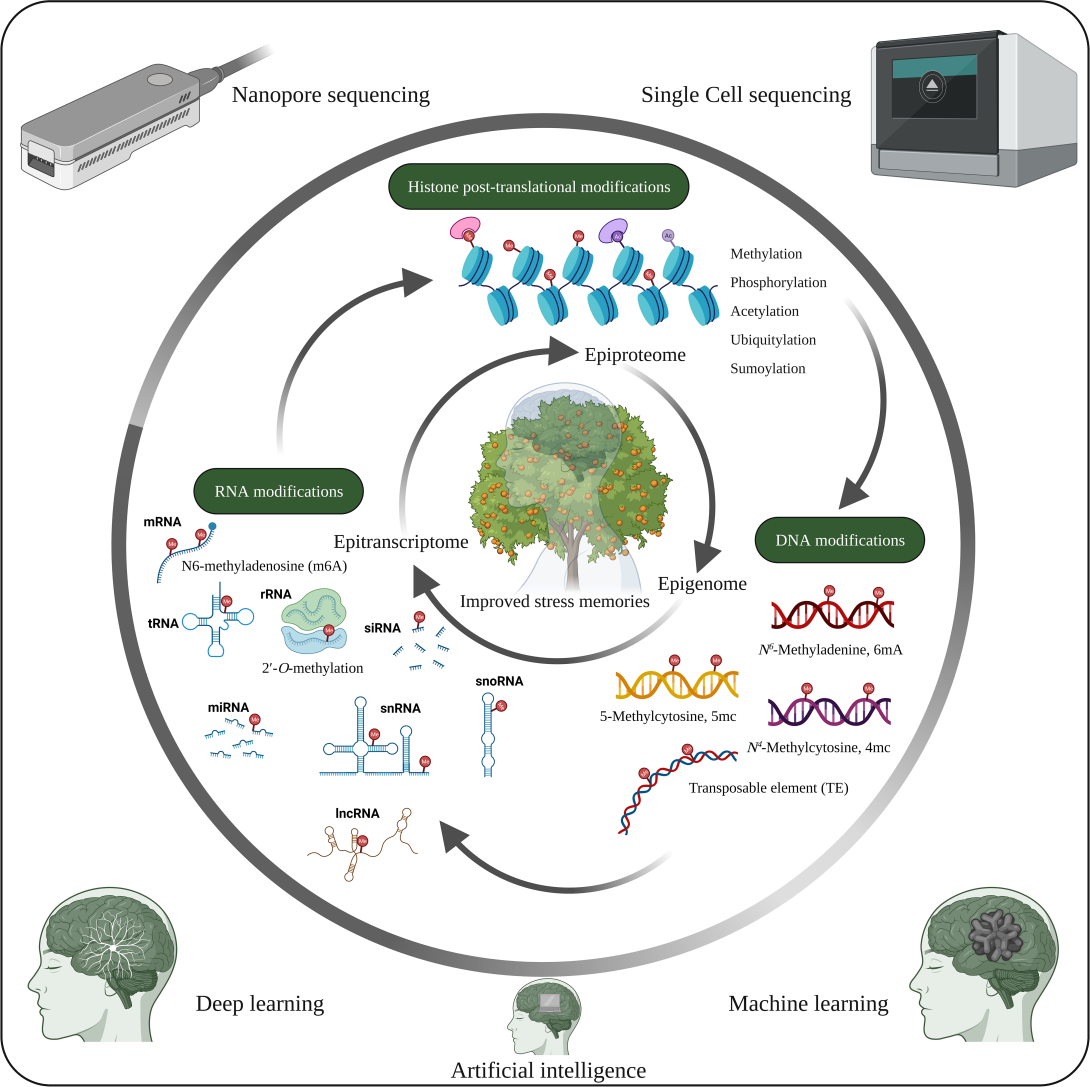
Future opportunities in plant stress memory. Cellular functions and stress tolerance depend on DNA, RNA and histone modifications, which are closely interconnected and lead to different phenotypic traits. However, the functions of the tissue-specific epigenome, epitranscriptome and epiproteome with respect to stress memory have not yet been extensively studied. Therefore, studies on the tissue-specific epigenome, epitranscriptome and epiproteome, along with recent developments such as artificial intelligence, machine learning, deep learning, nanopore sequencing and single-cell sequencing, could help identify potential mechanisms underlying stress memory. The image was created using BioRender.com.

Recent epigenetic modifications (**Figure 4**) such as 6mA in DNA (Li et al., 2022), epitranscriptomics (RNA modification) such as N^6^-methyladenosine (m^6^A), 5-methylcytosine (m^5^C), 5‐hydroxymethylcytosine (hm^5^C), N^7^-methylguanosine (m^7^G), Pseudouridine, 2′-*O*-methylation etc. (Hu et al., 2022; Murakami and Jaffrey, 2022; Ramakrishnan et al., 2022) play essential roles in plant development and stress response. Therefore, RNA modification is a new avenue for studying stress memory. Similarly, PTMs of proteins (epiproteome) (**Figure 4**) are key regulators of gene and protein activity (Song et al., 2020). Consequently, deciphering the epigenetic codes of stress memory requires future studies with advances in high-throughput sequencing and chromatin profiling technologies. The combined studies of epigenome, epitranscriptome and epiproteome of the specific cell and tissue on stress memory genes and their effects on epigenetic changes will greatly improve the understanding of epigenetic stress memory in specific cells and tissues. In addition, advances in machine learning, deep learning algorithms and big data analytics could help identify new epigenetic stress memories.

## Author contributions

MR, ZZ, and QW planned, designed and wrote the review. MR, SM, QW, ZZ, RK, and MZ outlined and edited the review. MR drew the images. MR, RK, ZA, MZ, SM, AS, GL, ZZ, and QW edited and revised the review. All authors contributed to the article and approved the submitted version.
